# *Bartonella* spp. - a chance to establish One Health concepts in veterinary and human medicine

**DOI:** 10.1186/s13071-016-1546-x

**Published:** 2016-05-10

**Authors:** Yvonne Regier, Fiona O´Rourke, Volkhard A. J. Kempf

**Affiliations:** Institute for Medical Microbiology and Infection Control, University Hospital, Goethe-University, Frankfurt am Main, Germany; Institut für Medizinische Mikrobiologie und Krankenhaushygiene, Klinikum der Goethe-Universität, Paul-Ehrlich-Str. 40, D-60596 Frankfurt am Main, Germany

**Keywords:** Ticks, Fleas, Lice, Cats, Dogs, Humans, Infection, Transmission, Zoonosis

## Abstract

Infectious diseases remain a remarkable health threat for humans and animals. In the past, the epidemiology, etiology and pathology of infectious agents affecting humans and animals have mostly been investigated in separate studies. However, it is evident, that combined approaches are needed to understand geographical distribution, transmission and infection biology of “zoonotic agents”. The genus *Bartonella* represents a congenial example of the synergistic benefits that can arise from such combined approaches: *Bartonella* spp. infect a broad variety of animals, are linked with a constantly increasing number of human diseases and are transmitted via arthropod vectors. As a result, the genus *Bartonella* is predestined to play a pivotal role in establishing a One Health concept combining veterinary and human medicine.

## Background

The threat of infectious diseases to mankind has never been greater than today. For the first time, political leaders of the 41^st^ “G7 summit” in Schloss Elmau/Germany on June 7–8, 2015, set the topic “global health” (including infectious diseases) as one of the key issues on their agenda. In the past, health issues played only a minor part in such international economic summits. However, governments have now realized that public health is an essential prerequisite for education, working capacity and therefore the economic prosperity of societies.

In this regard, it is important to recognize that human health and animal health are closely linked. An estimated 75 % of emerging infectious diseases are zoonotic and 28 % vector-borne [1]. Global warming represents an additional factor promoting the spread of these diseases as the geographic range of some vectors and reservoir hosts expands in response to a changing climate [2].

To respond to these challenges, the One Health concept aims to establish interdisciplinary collaborations between medical, veterinary and environmental researchers as well as public health officials for the early detection of health hazards affecting both humans and animals and to fight them on multiple levels. The genus *Bartonella* represents a prototypical example for zoonotic pathogens as *Bartonella* species are infectious agents for humans and animals. High pathogen prevalence and severe courses of infection raise the importance to investigate possible routes of transmission and to combat infections.

## The genus *Bartonella*: a diverse and expanding group of bacteria

The bacterial genus of *Bartonella* is comprised of Gram-negative, slow growing and facultative intracellular pathogens that infect mainly mammalian hosts and are often transferred via blood sucking arthropod vectors. *Bartonella* infections of humans and animals are often characterized by an intraerythrocytic bacteremia. At least 20 species are known to cause host-specific intraerythrocytic infections in their specific mammalian reservoir hosts, including the human-specific pathogens *Bartonella quintana* and *Bartonella bacilliformis,* the agents of trench fever and Oroya fever, respectively. A secondary tissue phase can be associated with development of vasculoproliferative lesions, e.g. bacillary angiomatosis (*Bartonella henselae, B. quintana*) or verruga peruana (*B. bacilliformis*) and may play a role in various other dermal conditions [3–7].

Molecular epidemiology techniques have revealed a remarkable diversity within the genus *Bartonella*. A wide variety of *Bartonella* spp. specialized to various mammalian hosts and transferred by specific arthropod vectors have been identified over the years and the prevalence of infection appears to be widespread across species and geographic regions. At least 13 species of *Bartonella* have been identified as pathogenic to humans with three species responsible for most of the clinically relevant infections in humans: *B. bacilliformis*, *B. quintana* and *B. henselae* [6].

*Bartonella* spp. infections are often chronic or asymptomatic in their reservoir hosts. Bacteria have been shown to infect erythrocytes, endothelial cells, macrophages and even human stem cells [8–17]. The infection of erythroctyes is host-specific and mediated by the so-called “Trw”-type 4 secretion system which facilitates host-restricted adhesion to erythrocytes [18]. Localized tissue manifestations may occur in reservoir and incidental hosts and the growth of bacteria in vascular tissue can lead to angioproliferative tumors and inflammation [5, 6, 12, 19]. The ability of *Bartonella* spp. to persist within immunoprivileged intracellular habitats is probably a key factor contributing to the establishment of chronic infections; however, the cyclic release of bacteria to the blood stream or the hemolytic activity of some species can also result in dramatic illnesses such as trench fever or Oroya fever, respectively [20]. The presence of *Bartonella* spp. in the blood stream of infected hosts or within the erythrocytes also facilitates their transfer via ingestion along with the blood meal of arthropod vectors [5, 21].

## *Bartonella* spp. infections in animals

### Cat infections

Cats are the main reservoir host for the species *B. henselae, B. clarridgeiae* (both of which can cause cat scratch disease) and *B. koehlerae* (a causative agent of endocarditis in humans) [22–24]. Infected cats are often clinically asymptomatic although they suffer from relapsing bacteremia over long periods of time [25]. Co-infections with more than one *Bartonella* species are not uncommon [26–29].

Transmission of *Bartonella* spp. among cats occurs via arthropod vectors, predominantly fleas. Uninfected cats kept together with infected cats in a specific ectoparasite-free environment do not become seropositive emphasizing the importance of arthropod vectors in the transmission of disease. Furthermore, the transmission via arthropod vectors appears to be essential as no direct transmission of *B. henselae* from cat to cat has been documented experimentally and flea-prevention measures have been shown to be effective in preventing pathogen transmission [30–35]. Prevalence of infection is highest in warm, moist areas with a higher ectoparasite burden (0 % in Norway *versus* 68 % in the Phillipines) [8, 22, 30, 36–38]. Up to 50 % of all cats (stray and pet) living in regions where fleas are endemic, harbor bacteremic *Bartonella* infections [26, 28]. Usually, cats are bacteremic for weeks or months but even longer infection intervals are possible. Young cats are more likely to be bacteremic than old cats and stray cats more than pet cats [8, 22, 23]. Cats were tested in several regions of Spain, for *B. henselae* seroreactivity and 50 % were shown to be positive. It is known, however, that serum antibodies have a limited value for the detection of active infections. In the same study, DNA of *Bartonella* spp. was detected in 4.4 % of the examined cat fleas [39]. *Bartonella* spp. have also been isolated from cat blood in various other locations throughout the world (e.g. from San Francisco/USA, North Carolina/USA, Hawaii/USA, Japan, Sydney, New Zealand, the Netherlands, France, Indonesia and Germany) [26, 28, 40–49].

Those *Bartonella* strains which have been isolated from healthy cats were usually not of the same genetic background as those strains detected from infected humans. Some of the feline strains were never found on patients and therefore might be less pathogenic for humans [50, 51].

Although healthy cats can be infected with *B. henselae* and *B. clarridgeiae* for months or even years, there is evidence that cats may also suffer from the persistent infection [26]. Especially infections with *Bartonella* spp. that are not believed to be specifically adapted to the cat as reservoir host (e.g. *Bartonella vinsonii* subsp. *berkhoffii*) can result in more serious clinical symptoms, e.g. osteomyelitis [30, 31, 52]. Several seroepidemiological studies indicated a correlation between seroreactivity and stomatitis, kidney- and urinary tract-diseases and uveitis [26, 53–55]. Another survey found that stomatitis is associated with the detection of *Bartonella* spp. but not with seroreactivity and revealed no association with uveitis, neurological symptoms and chronic kidney diseases; however, a weak association between seroreactivity and idiopathic feline lower urinary tract disease was detected [56]. Cats experimentally infected with *B. henselae* or *B. clarridgeiae* were found to suffer from fever, eosinophilia, lymphadenomegaly and anemia. Perinatal transmission was not described but reproductive disorders were observed. Furthermore, some cats suffered from transient neurological disorders, endocarditis and focal myocarditis [26, 57–62].

Isolation of *Bartonella* spp. has been possible from cats whose owners suffered from cat scratch disease and bacillary angiomatosis and *B. clarridgeiae* was isolated from a kitten that had caused cat scratch disease in a veterinarian [25, 26, 40, 63]. *Bartonella quintana* was found in the mouth of a domestic cat and there are reported cases of humans suffering from *B. quintana* infections where no louse infestation was verifiable but contact with cats was reported [30, 64]. In one case *B. qintana* was detected in a woman and two cats, one of which had bitten the woman previously providing further evidence for the incidental zoonotic transmission of *Bartonella* between animals and humans [65]. Antimicrobial treatment for pathogen eradication in cats is not broadly recommended; therefore, ectoparasite-control (e.g. collars containing acaricides) is crucial as the main instrument to lower the *Bartonella* prevalence in cats and therefore reduces the risk of pathogen-transmission to humans [30, 35].

## Dog infections

Dogs represent an incidental host for *Bartonella* and two species are known to cause clinically apparent infections: *B. vinsonii* subsp. *berkhoffii*, causing endocarditis, arrhythmias, myocarditis, granulomatous lymphadenitis and granulomatous rhinitis, and *B. henselae* causing peliosis hepatis [66–71]. In a study from the United States surveying *Bartonella* bacteremia in dogs, *B. henselae* was found in 30 of 61 infected dogs [72]; however, there are also rare cases in which other *Bartonella* spp. have caused disease in dogs: *B. clarridgeiae*, *B. washoensis* and *B. quintana* were isolated from dogs suffering from endocarditis [8, 22, 23]. To date, all *Bartonella* spp. identified in sick dogs are also known as pathogenic or potentially pathogenic infectious agents for humans and this observation led to the suggestion that dogs might act as useful sentinel species and important comparative models for human infections [22, 73].

Domestic dogs are generally incidental hosts for *B. henselae* with a reported seroprevalence of ~10 % in healthy dogs in the United States and ~27 % in sick dogs [30, 74]. Similar to cat epidemiology, seroprevalence increases in warmer regions [30]. *Bartonella henselae*, *B. quintana*, *B. vinsonii* subsp. *berkhoffii* and *B. bovis* have been detected in mouth swabs from dogs and there is some evidence that dogs may be able to transmit *B. henselae* to humans via bites [22, 30, 37, 75]. Because of the prolonged bacteremia of *B. vinsonii* subsp. *berkoffii* in dogs, they are suspected to represent the reservoir host of these bacteria and seroreactivity of dogs against *B. vinsonii* subsp. *berkoffii* is found worldwide [22, 26, 32]. In Gabon, *B. clarridgeiae* was also isolated from ~2 % of the examined dogs indicating that these animals may represent a potential reservoir host for *Bartonella* spp. in Africa [76].

Serological surveys suggest that *B. vinsonii* subsp. *berkhoffii* may also cause immuno-mediated hemolytic anemia, neutrophil or granulomatous meningoencephalitis, neutrophil polyarthritis and uveitis in dogs [8, 22]. *Bartonella vinsonii* subsp. *berkhoffii* can cause endocarditis, especially in large breed dogs with a predisposition for aortic valve involvement. Intermittent lameness and fever of unknown origin can occur several months before endocarditis. Myocarditis without an associated endocarditis is also possible and may result in arrhythmias, syncope or sudden death [26, 67]. To detect *Bartonella* spp. as the causative agent of infectious endocarditis in dogs, diagnostic PCRs should be performed from blood or heart valve specimen as blood cultures often remain negative [77, 78]. High antibody-titers and characteristic lesions in echocardiography are also suspicious for *Bartonella* endocarditis. In most cases, *Bartonella* infect the aortic valve causing aortic insufficiency leading to severe chronic heart failure and arrhythmias [66–68, 77, 79, 80].

## Infections of other mammals

There are many publications describing *Bartonella* infections of numerous mammals and even reptiles. For instance, *Bartonella* spp. have been detected in a wide variety of wild and domestic animals throughout the world including, e.g. mountain lions, bobcats, coyotes, grey foxes, elks, mule deer, cougars, rabbits, several rodent species, cattle, belugas, bats and porpoises*.* However, it is unclear which diseases if any are associated with such infections and if these animals play a role as potential reservoir hosts. [26, 30, 81–92].

## *Bartonella* spp. infections of humans

The first human pathogenic *Bartonella* species to be identified in the early 1900s was *B. bacilliformis*. This human-specific bacterium causes a biphasic disease characterised by a primary hemolytic fever (“Oroya fever”) with high mortality (up to 90 %), followed by a chronic vasculoproliferative tissue phase (“verruga peruana”). Pathogens are transmitted by the sand fly (*Lutzomyia verrucarum*). The human body louse (*Pediculus humanus humanus*) transmits *B. quintana*, a second human pathogenic *Bartonella* species which emerged as a major agent of disease causing debilitating cyclic fever (“trench fever“) during World War I. Today, trench fever occurs mainly in the homeless population or among drug addicts. Endocarditis, generalized lymphadenopathy and bacillary angiomatosis are symptoms of *B. quintana* infections in immunocompromised people [93–100].

Of the three most significant human pathogenic *Bartonella* species *B. henselae* is the most common symptomatic infection causing agent identified in the modern clinical setting. *Bartonella henselae* infection is the cause of multiple clinical entities in humans and infections result in differential disease outcomes often depending on the immune status of the patient. Humans become infected via the scratches or bites of infected cats contaminated with infected flea feces or are directly contaminated with infected blood. Dogs are also suspected to be an additional reservoir for *B. henselae* transmission to humans [41]. In immunocompetent patients, infections normally cause cat scratch disease which is often self-limiting with no need for antibiotic treatment. Typically, two to three weeks after infection, a unilateral lymphadenitis in the draining region of the lymph node near the site of inoculation can be observed. In ~10 % of the cases, the lymph node forms a fistula where pus is draining. Other symptoms include chronic lymph node swelling, fever, headache, skin and mucosal lesions near the inoculation site and splenomegaly. “Blood-culture negative”-endocarditis, oculoglandular involvement (“Parinaud’s syndrome”), encephalopathy, neuroretinitis and osteomyelitis are described as complications of infection [101]. Recurring or systemic infections can be treated with macrolides. In immunocompromised hosts, chronic infections can occur, leading to angioproliferative diseases like bacillary angiomatosis and peliosis hepatis which can be fatal if not treated [6, 19].

Several *Bartonella* spp. have been reported as cause of fever of unknown origin and culture-negative endocarditis in humans and animals [102–105]. In humans, endocarditis caused by *B. henselae*, *B. quintana*, *B. elizabethae*, *B. vinsonii* subsp. *berkhoffii*, *B. vinsonii* subsp. *arupensis*, *B. koehlerae*, *B. alsatica*, *B. washoensis* and Candidatus *B. mayotimonensis* have been reported [24, 106–114]; however, human endocarditis cases are most often associated with *B. henselae* and *B. quintana* [79, 80, 115]. In most cases, high anti-*Bartonella*-IgG antibody-titers can be detected [102, 116].

Co-infections with more than one *Bartonella* spp. (even in immunocompetent patients) [117–119] and with other zoonotic bacterial species have been reported. Co-infections with *Borrelia burgdorferi* (*sensu lato*) and *B. henselae* were described in patients with atypical neuroborreliosis [120–122]. Furthermore, surveys showed the occurrence of co-infections with *B. henselae* in people suffering from persistent symptoms after borreliosis treatment where ticks might have been the source of infection [121]. The transmission of multiple pathogens via co-infected vectors might contribute to atypical disease progression and should be considered for the diagnosis of tick-borne diseases [121, 123, 124]. However, it must be stated that the occurrence of chronic, atypical tick-borne co-infections in patients with chronic, nonspecific illnesses is highly controversially discussed. As reviewed by Lantos & Wormser, in most reported cases of *Bartonella* and *Borrelia* co-infections, laboratory diagnostics were not properly performed [125].

Different population groups are exposed to animals and arthropod vectors in variable dimensions. In particular, veterinarians, veterinary technicians or zookeepers might be at increased risk of infection with *Bartonella* spp. [119, 126]. For example, one case of *B. vinsonii* subsp. *berkhoffii*, transmission to a veterinarian was likely caused by a needle puncture injury [127]. *Bartonella* infections have even been suspected to have been a contributing factor in the death of two veterinarians in 2013 [128]. In an epidemiological study, *Bartonella* DNA was also detected in the blood of 28 % of veterinary workers whereas no *Bartonella* was detected in control subjects [126]. The prevalence of *Bartonella* infections has also been found to be elevated in other risk groups. In a recent study in Germany *B. henselae* IgG antibodies were found in ~45 % of forestry workers which may be due to a higher contact with arthropods which is inevitable during forest work [129]. From a One Health perspective, the identification of possible vectors and means of *Bartonella* transmission is crucial to reduce occupational hazards in certain risk groups and prevent such cases of *Bartonella* transmission in future.

Blood transfusion has also been identified as a risk factor for the transmission of *Bartonella* infections. The transmission of infection via blood transfusion was first shown 20 years ago in cats [58] and a very recent study from Brazil has also indicated a ~3 % prevalence of *Bartonella* spp. in asymptomatic human blood donors. Remarkably, the results of this study found that professionals with animal contact were seven times more likely to harbor *Bartonella* than other blood donors and individuals with cat contact or a history of tick bite were three to four times more likely to be infected with *Bartonella* spp. [130]. Considering that patients receiving blood transfusions are already in a state of weakened health, screening of blood donors for *Bartonella* infections, especially in certain risk groups should be considered to prevent transmission of infection.

## Vector transmission of *Bartonella* spp.

The transmission cycle of bartonellosis is typical for vector-borne diseases. Typically, infections are characterized by persistent intraerythrocytic bacteremia within the reservoir host. Infected blood is ingested by the blood sucking arthropod vector and subsequently transmitted to a further reservoir or incidental host. To date, vector-competence of several arthropods has been proven for *Bartonella* spp. transmission and additional vectors competencies are suspected in many more.

## Flea transmission of *Bartonella* spp.

The cat flea (*Ctenocephalides felis*) represents the main vector for *B. henselae* infection among cats. Its vector-competence for *B. henselae* transmission is experimentally proven and its presence is essential for the maintenance of *B. henselae* infection within the cat population. The contamination of the flea-feeding wound, or other wounds such as scratches or bites with contaminated flea feces has been identified as an important transmission route among hosts including cats and humans [30, 33, 96, 131–133]. Bacteria are replicating in the flea’s intestine and are secreted with the feces over the life-span of the flea (~12 days). The excreted flea feces contain *B. henselae* within 24 h of a blood meal [134].

Further supporting the importance of the flea as a vector of *B. henselae* transmission, epidemiological studies have shown an increased risk of *B. henselae* infection in cats suffering from flea infestation and the use of flea-prevention collars has also been shown to be effective in preventing the transmission of *B. henselae* infection from cat to cat [35]. Once infected, *B. henselae* bacteremia in cats can last for weeks, months or even longer than one year supporting further vector-transmission [22, 23, 29, 39, 132, 135, 136]. In addition to *B. henselae*, cats are susceptible to infections with *B. quintana*, *B. koehlerae*, *B. clarridgeiae*, *B. vinsonii* subsp. *berkhoffii* and *B. bovis* which have also been detected in cat fleas. With the exception of *B. bovis*, these species can also be pathogenic to humans [6, 30, 52, 137]. Flea control is highly recommended in endemic areas to reduce the pathogen exposition of cats and humans [39].

In addition to the cat flea various other flea species may also play important roles in *Bartonella* transmission. *Bartonella* spp. were detected in several flea species collected from bats and different rodents [30, 96, 138–144]. Vector-competence, however, has not been confirmed experimentally for these species.

## Louse transmission of *Bartonella* spp.

The human body louse (*Pediculus humanus humanus*) represents the vector of human to human-transmission of *B. quintana*. Environmental factors supporting louse infestation such as unhygienic living conditions lead to an increased risk of infection. In the past, infections with *B. quintana* were a severe medical problem in the trenches and in prisoners of war camps of World War I from which the name “trench fever” arises. Today, mostly homeless people or drug addicts are affected resulting in the term “urban trench fever” [99, 145]. The vector becomes infected when adult lice feed on bacteremic hosts. *Bartonella quintana* reaches the louse gut and can infect humans when bite sites or other wounds are contaminated with infected louse feces [30, 64, 65, 96, 97, 146]. *Bartonella* spp. have also been detected from several other louse species (e.g. *Neohaematopinus sciuri*, *Hoplopleura sciuricola, Pediculus humanus capitis* and others) which may also serve as vectors [96, 139, 147, 148].

## Sand fly transmission of *Bartonella* spp.

The sand fly (*Lutzomyia verrucarum*) transmits *B. bacilliformis* from humans to humans and its vector-competence has been proven experimentally [96, 98, 149–151]. The occurrence of the disease is strictly limited to the Peruvian Andes where the vector is endemic. However, it should be considered that climate change may extend the distribution area of this vector and thereby increase the spread of *B. bacilliformis*.

## Tick transmission of *Bartonella* spp.

**Fig. 1:**
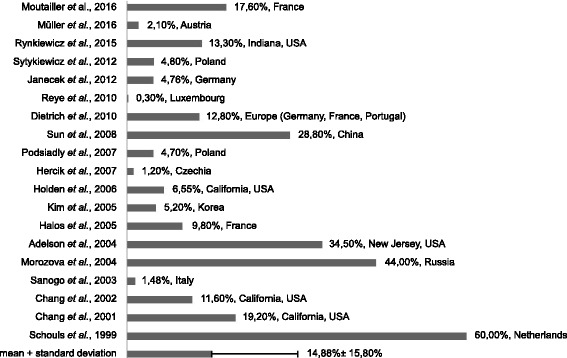
Percentage of ticks found to harbor *Bartonella* spp. DNA (literature review). Presence of *Bartonella* spp. was determined via molecular techniques. Overall, *Bartonella* spp. were found in ~14.88 ± 15.80 % of ticks. Countries in which ticks were collected are depicted within the diagram

Ticks are known to act as vectors for many different bacterial, protozoan and viral pathogens. Hard ticks (e.g. *Ixodes* spp., *Dermacentor* spp.) usually feed three times during their life-cycle and can possibly be infected with different pathogens during every blood meal. Hosts can be bitten by ticks several times during their lifetime presenting multiple opportunities for pathogen transmission [152–154]. Several studies have detected the presence of *Bartonella* spp. in various tick species from around the world [26, 84, 93, 120, 121, 123, 138, 152, 155–168]. The prevalence of *Bartonella* DNA in hard ticks in Europe has been shown to be as high as 40 % [158]. In a recent study conducted in Finland, ticks were found to contain no detectable *Bartonella* DNA whereas DNA of *Borrelia* spp. was found frequently at ~ 19 % [169]. On the other hand, *Bartonella* DNA was detected in ~2 % of ticks collected in a recent study from Austria [170]. Figure 1 displays the percentage of ticks found to harbor *Bartonella* in different studies. Overall, in ~15 % of ticks studied *Bartonella* DNA was detectable.

Vector competence has been demonstrated experimentally by the use of artificial tick-feeding procedures for *B. henselae* [171] and a murine *B. birtlesii* infection model [172]; however, vector competence of naturally infected ticks has still not been confirmed.

*Bartonella* DNA has been detected in hard ticks removed from dogs. However, as DNA was detectable in only some but not all ticks removed from one particular dog, the infection of the tick may have been acquired from another source previously [173]. Furthermore, several studies indicate co-transmission of *Bartonella* with other tick-borne pathogens (e.g. *Ehrlichia, Babesia*) in dogs [66, 174–178]. In a study surveying dogs with endocarditis from California, all dogs that were infected with *Bartonella* were also seroreactive to *Anaplasma phagocytophilum,* another tick-borne pathogen [77].

In two cases, *B. henselae* DNA was detected in ticks collected from the home of patients which suffered from Lyme disease and which did not respond to a *Borrelia-*specific antibiotic therapy. In another study, *Bartonella* DNA was detectable in human blood after tick bites and recently, *B. henselae* and three other animal-associated *Bartonella* species (*B. doshiae, B. schoenbuchensis* and *B. tribocorum*) were isolated from patients suffering from undifferentiated chronic illness and who had reported tick bites [121, 179, 180].

Several case reports of *B. henselae* infections of humans have been published where no or very limited cat contact was reported, limiting the possibility of transmission via cats or cat fleas. Authors concluded that transmission via arthropod vectors (e.g. ticks) may provide an alternative explanation [96, 181].

The most important reservoir hosts for tick-borne pathogens are small rodents as they are the preferred hosts of tick larvae and nymphs. Several *Bartonella* spp. have been detected in these small mammals further supporting the possibility that ticks may represent a vector for *Bartonella* transmission [84, 87, 152–154, 162, 182, 183]. *Bartonella* spp. have also been isolated from cattle and mule deer in North America. As ruminants are rarely infested with fleas, ticks seem to be more likely to transmit these pathogens to these animals [81].

Nevertheless, it must be mentioned that the transmission of *Bartonella* spp. via ticks to humans and animals is still controversially discussed. Clearly, *Bartonella* DNA which was found in several tick species in multiple studies does not prove the presence of viable bacteria. Therefore, some researchers doubt heavily that *Bartonella* spp. are transmitted by ticks [125, 184]. Furthermore, concerns about the relevance of experimental tick transmission studies performed with an artificial feeding system [171] have been raised [184]: the amount of colony forming units in the blood was criticized to be much higher than it would be in natural infected bacteremic cats and the *B. henselae* strain that was used is not representative for *Bartonella* strains found in nature [184]. Building on these points, the authors conclude that neither of these studies demonstrated transmission of *Bartonella* spp. from ticks to mammalian hosts [125]. At least for *B. birtlesii*, tick transmission was proven in a murine infection model [172] whereas a *bona fide* tick transmission of *B. henselae* has not been demonstrated so far.

## The role of other arthropods in the transmission of *Bartonella* spp.

*Bartonella* species have been found in biting flies collected from cattle in California: *B. bovis* was detected in a horn fly (*Haematobia* spp.) and *B. henselae* was found in a stable fly (*Stomoxy* spp.) [185]. Several studies found *Bartonella* spp. in mites collected from rodents and bats from Korea, Egypt and Costa Rica [96, 162, 186]. Deer keds (*Lipoptena mazamae* and *Lipoptena cervi*) were shown to be infected with *B. henselae* and *B. schoenbuchensis* [30, 187–189]. *Lipoptena* species usually feed on deer but were also found on horses, cattle and humans. *Bartonella schoenbuchensis* was detected in *Lipoptena cervi* from a deer (*Capreolus capreolus*) in Germany and is suspected to be the causative agent of deer ked dermatitis in humans [190]. *Bartonella* was also found in several other species of the family *Hippoboscidae* indicating that they may play a role in the transmission of these bacteria [96, 191]. However, no experimental transmission studies with these species have been performed nor do any data exist on the transmission of *B. schoenbuchensis* by *Lipoptena* spp. to humans.

## The need for scientific One Health approaches in *Bartonella* research

**Fig. 2:**
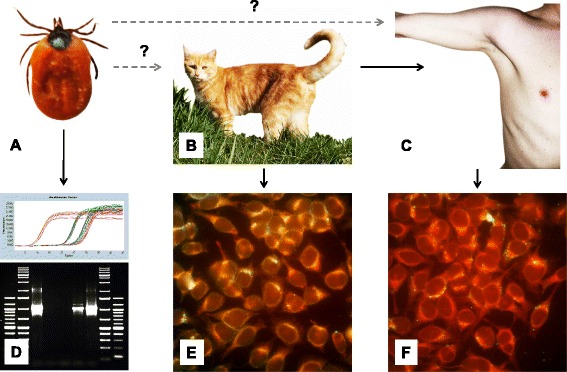
One Health concept for detecting of *Bartonella* infections in humans and domestic animals. Fleas transmit *B. henselae* to cats. Transmission of *B. henselae* by ticks, e.g. *Ixodes ricinus* **a** to cats **b** or humans **c** is assumed but controversially discussed. *Bartonella* DNA can be detected in ticks via real-time PCR or conventional PCR **d**. Finally, *B. henselae* infections of cats and humans can be diagnosed by indirect immunofluorescence analysis (anti-B. henselae-IgG: green, **e**, **f**)

When discussing transmission of *Bartonella* spp. from animals to humans, e.g. via arthropod vectors, a more integrative approach elucidating *Bartonella* prevalence in vectors as well as the infection status of animals and humans would clearly help to increase understanding of *Bartonella* infection dynamics, infection risk and prevent speculative and non-evidence-based conclusions. Such an approach might, for instance, include the investigation of the prevalence of *Bartonella* DNA or (even more reliable) of viable *Bartonella* species in feeding ticks, combined with parallel detection of these pathogens via direct detection or seroprevalence in animals (e.g. pets) and humans (e.g. pet owners). Figure 2 shows this concept of such a One Health approach.

For instance, a coordinated set of data might include (i) the pathogen-DNA status of questing ticks (with bacterial, e.g. *Bartonella* 16S-rDNA sequences; analyzed by conventional PCRs or metagenomics analysis), (ii) the direct detection of these pathogen(s) or detection of pathogen-specific (e.g. *Bartonella*) antibodies in animals (arguing for infection of the pet), and (iii) the direct detection of these pathogen(s) if feasible or the determination of the respective pathogen-specific antibodies in humans (e.g. pet owner) in parallel (arguing for a previous or present infection).

First incidental results from a clinical case revealed interesting findings: In a female, adult, half-engorged *I. ricinus* tick (black forest, Germany) which was feeding for ~1–2 days on a cat, *Bartonella*-DNA was detected via nested-PCR. Sequence analysis revealed most likely the presence of *B. henselae* 16S-rDNA (99 % sequence homology). For medical reasons, a serum sample from the cat was taken (because of unspecific illness). Immunofluorescence testing revealed cat antibody titers of 1:640 whereas no specific *B. henselae*-antibodies were detected in the pet owner. This setting might be interpreted as follows: The questing *I. ricinus* tick was probably ingesting *B. henselae*-containing cat blood as anti-*B. henselae* IgG antibodies were detectable. The pet owner, however, had no serological evidence for having been exposed to *B. henselae*. As a further option, a chronic *B. henselae* infection of the cat might additionally be confirmed (e.g. via PCR-analysis of peripheral blood). The application of such One Health approaches in prospective scientific studies would be useful to assess the real risk of transmission of *Bartonella* spp. from pets to pet owners and to clarify the role of ticks in this process.

## Conclusions

The cumulative data collected in many studies and conducted in several countries throughout the world indicate that infections with *Bartonella* spp. might represent an underappreciated danger for human and animal health. A great deal more research is needed to specify arthropod vectors and characteristics of diseases caused by *Bartonella* species. To date, strict ectoparasite-control is highly recommended to lower the risk of *Bartonella* infection from arthropod vectors to domestic animals and pets, thereby preventing pathogen transmission from animals to human owners. Overall, these results demonstrate that reliable data about vector transmission of *Bartonella* spp. from animals to humans can only be generated through the application of scientific One Health approaches which take into account the epidemiological factors and interactions of humans, animals and their environments as an integrated system.

## Abbreviations

DNA: deoxyribonucleic acid
PCR: polymerase chain reaction

## References

[1] Taylor LH, Latham SM, Woolhouse ME (2001). Risk factors for human disease emergence. Philos Trans R Soc Lond B Biol Sci.

[2] Mills JN, Gage KL, Khan AS (2010). Potential influence of climate change on vector-borne and zoonotic diseases: a review and proposed research plan. Environ Health Perspect.

[3] Anderson BE, Neuman MA (1997). *Bartonella* spp. as emerging human pathogens. Clin Microbiol Rev.

[4] Maurin M, Birtles R, Raoult D (1997). Current knowledge of *Bartonella* species. Eur J Clin Microbiol Infect Dis.

[5] Dehio C (2005). *Bartonella*-host-cell interactions and vascular tumour formation. Nat Rev Microbiol.

[6] Kaiser PO, Riess T, O’Rourke F, Linke D, Kempf VAJ (2011). *Bartonella* spp.: throwing light on uncommon human infections. Int J Med Microbiol.

[7] Rossi MA, Balakrishnan N, Linder KE, Messa JB, Breitschwerdt EB (2015). Concurrent *Bartonella henselae* infection in a dog with panniculitis and owner with ulcerated nodular skin lesions. Vet Dermatol.

[8] Boulouis HJ, Chang CC, Henn JB, Kasten RW, Chomel BB (2005). Factors associated with the rapid emergence of zoonotic *Bartonella* infections. Vet Res.

[9] Kempf VA, Schaller M, Behrendt S, Volkmann B, Aepfelbacher M, Cakman I (2000). Interaction of *Bartonella henselae* with endothelial cells results in rapid bacterial rRNA synthesis and replication. Cell Microbiol.

[10] Kempf VA, Volkmann B, Schaller M, Sander CA, Alitalo K, Riess T (2001). Evidence of a leading role for VEGF in *Bartonella henselae*-induced endothelial cell proliferations. Cell Microbiol.

[11] Mändle T, Einsele H, Schaller M, Neumann D, Vogel W, Autenrieth IB (2005). Infection of human CD34+ progenitor cells with *Bartonella henselae* results in intraerythrocytic presence of *B. henselae*. Blood.

[12] O’Rourke F, Mändle T, Urbich C, Dimmeler S, Michaelis UR, Brandes RP (2015). Reprogramming of myeloid angiogenic cells by *Bartonella henselae* leads to microenvironmental regulation of pathological angiogenesis. Cell Microbiol.

[13] Maeno N, Oda H, Yoshiie K, Wahid Fujimura T, Matayoshi S (1999). Live *Bartonella henselae* enhances endothelial cell proliferation without direct contact. Microb Pathog.

[14] Resto-Ruiz SI, Schmiederer M, Sweger D, Newton C, Klein TW, Friedman H (2002). Induction of a potential paracrine angiogenic loop between human THP-1 macrophages and human microvascular endothelial cells during *Bartonella henselae* infection. Infect Immun.

[15] Kyme PA, Haas A, Schaller M, Peschel A, Iredell J, Kempf VAJ (2005). Unusual trafficking pattern of *Bartonella henselae*-containing vacuoles in macrophages and endothelial cells. Cell Microbiol.

[16] Schülein R, Guye P, Rhomberg TA, Schmid MC, Schröder G, Vergunst AC (2005). A bipartite signal mediates the transfer of type IV secretion substrates of *Bartonella henselae* into human cells. Proc Natl Acad Sci U S A.

[17] Lu YY, Franz B, Truttmann MC, Riess T, Gay-Fraret J, Faustmann M (2013). *Bartonella henselae* trimeric autotransporter adhesin BadA expression interferes with effector translocation by the VirB/D4 type IV secretion system. Cell Microbiol.

[18] Vayssier-Taussat M, Le Rhun D, Deng HK, Biville F, Cescau S, Danchin A (2010). The Trw type IV secretion system of *Bartonella* mediates host-specific adhesion to erythrocytes. PLoS Pathog.

[19] Koehler JE, Quinn FD, Berger TG, LeBoit PE, Tappero JW (1992). Isolation of *Rochalimaea* species from cutaneous and osseous lesions of bacillary angiomatosis. N Engl J Med.

[20] Foucault C, Rolain JM, Raoult D, Brouqui P (2004). Detection of *Bartonella quintana* by direct immunofluorescence examination of blood smears of a patient with acute trench fever. J Clin Microbiol.

[21] Pulliainen AT, Dehio C (2012). Persistence of *Bartonella* spp. stealth pathogens: from subclinical infections to vasoproliferative tumor formation. FEMS Microbiol Rev.

[22] Chomel BB, Boulouis HJ, Maruyama S, Breitschwerdt EB (2006). *Bartonella* spp. in pets and effect on human health. Emerg Infect Dis.

[23] Chomel BB, Boulouis HJ, Breitschwerdt EB (2004). Cat scratch disease and other zoonotic *Bartonella* infections. J Am Vet Med Assoc.

[24] Avidor B, Graidy M, Efrat G, Leibowitz C, Shapira G, Schattner A (2004). *Bartonella koehlerae*, a new cat-associated agent of culture-negative human endocarditis. J Clin Microbiol.

[25] Kordick DL, Wilson KH, Sexton DJ, Hadfield TL, Berkhoff HA, Breitschwerdt EB (1995). Prolonged *Bartonella* bacteremia in cats associated with cat-scratch disease patients. J Clin Microbiol.

[26] Breitschwerdt EB, Kordick DL (2000). *Bartonella* infection in animals: carriership, reservoir potential, pathogenicity, and zoonotic potential for human infection. Clin Microbiol Rev.

[27] Gurfield AN, Boulouis HJ, Chomel BB, Heller R, Kasten RW, Yamamoto K (1997). Coinfection with *Bartonella clarridgeiae* and *Bartonella henselae* and with different *Bartonella henselae* strains in domestic cats. J Clin Microbiol.

[28] Heller R, Artois M, Xemar V, de Briel D, Gehin H, Jaulhac B (1997). Prevalence of *Bartonella henselae* and *Bartonella clarridgeiae* in stray cats. J Clin Microbiol.

[29] Kordick DL, Brown TT, Shin K, Breitschwerdt EB (1999). Clinical and pathologic evaluation of chronic *Bartonella henselae* or *Bartonella clarridgeiae* infection in cats. J Clin Microbiol.

[30] Mosbacher ME, Klotz S, Klotz J, Pinnas JL (2011). *Bartonella henselae* and the potential for arthropod vector-borne transmission. Vector Borne Zoonotic Dis.

[31] Chomel BB, Kasten RW, Floyd-Hawkins K, Chi B, Yamamoto K, Roberts-Wilson J (1996). Experimental transmission of *Bartonella henselae* by the cat flea. J Clin Microbiol.

[32] Kordick DL, Breitschwerdt EB (1998). Persistent infection of pets within a household with three *Bartonella* species. Emerg Infect Dis.

[33] Guptill L (2003). Bartonellosis. Vet Clin North Am Small Anim Pract.

[34] Bradbury CA, Lappin MR (2010). Evaluation of topical application of 10 % imidacloprid-1 % moxidectin to prevent *Bartonella henselae* transmission from cat fleas. J Am Vet Med Assoc.

[35] Lappin MR, Davis WL, Hawley JR, Brewer M, Morris A, Stanneck D (2013). A flea and tick collar containing 10 % imidacloprid and 4.5 % flumethrin prevents flea transmission of *Bartonella henselae* in cats. Parasit Vectors.

[36] Jameson P, Greene C, Regnery R, Dryden M, Marks A, Brown J (1995). Prevalence of *Bartonella henselae* antibodies in pet cats throughout regions of North America. J Infect Dis.

[37] Skerget M, Wenisch C, Daxboeck F, Krause R, Haberl R, Stuenzner D (2003). Cat or dog ownership and seroprevalence of ehrlichiosis, Q fever, and cat-scratch disease. Emerg Infect Dis.

[38] Solano-Gallego L, Hegarty B, Espada Y, Llull J, Breitschwerdt E (2006). Serological and molecular evidence of exposure to arthropod-borne organisms in cats from northeastern Spain. Vet Microbiol.

[39] Gracia MJ, Marcén JM, Pinal R, Calvete C, Rodes D (2015). Prevalence of *Rickettsia* and *Bartonella* species in Spanish cats and their fleas. J Vector Ecol.

[40] Koehler JE, Glaser CA, Tappero JW (1994). *Rochalimaea henselae* infection. A new zoonosis with the domestic cat as reservoir. JAMA.

[41] Keret D, Giladi M, Kletter Y, Wientroub S (1998). Cat-scratch disease osteomyelitis from a dog scratch. J Bone Joint Surg.

[42] Demers DM, Bass JW, Vincent JM, Person DA, Noyes DK, Staege CM (1995). Cat-scratch disease in Hawaii: etiology and seroepidemiology. J Pediatr.

[43] Maruyama S, Nogami S, Inoue I, Namba S, Asanome K, Katsube Y (1996). Isolation of *Bartonella henselae* from domestic cats in Japan. J Vet Med Sci.

[44] Branley J, Wolfson C, Waters P, Gottlieb T, Bradbury R (1996). Prevalence of *Bartonella henselae* bacteremia, the causative agent of cat scratch disease, in an Australian cat population. Pathol.

[45] Flexman JP, Lavis NJ, Kay ID, Watson M, Metcalf C, Pearman JW (1995). *Bartonella henselae* is a causative agent of cat scratch disease in Australia. J Infect.

[46] Joseph AK, Wood CW, Robson JM, Paul SL, Morris AJ (1997). *Bartonella henselae* bacteraemia in domestic cats from Auckland. N Z Vet J.

[47] Bergmans AM, de Jong CM, van Amerongen G, Schot CS, Schouls LM (1997). Prevalence of *Bartonella* species in domestic cats in The Netherlands. J Clin Microbiol.

[48] Marston EL, Finkel B, Regnery RL, Winoto IL, Graham RR, Wignal S (1999). Prevalence of *Bartonella henselae* and *Bartonella clarridgeiae* in an urban Indonesian cat population. Clin Diagn Lab Immunol.

[49] Arvand M, Klose AJ, Schwartz-Porsche D, Hahn H, Wendt C (2001). Genetic variability and prevalence of *Bartonella henselae* in cats in Berlin, Germany, and analysis of its genetic relatedness to a strain from Berlin that is pathogenic for humans. J Clin Microbiol.

[50] Breitschwerdt EB, Broadhurst JJ, Cherry NA. *Bartonella henselae* as a cause of acute-onset febrile illness in cats. J Feline Med Surg. 2015. doi:10.1177/2055116915600454. 10.1177/2055116915600454 PMC5362002 28491382

[51] Gil H, Escudero R, Pons I, Rodríguez-Vargas M, García-Esteban C, Rodríguez-Moreno I (2013). Distribution of *Bartonella henselae* variants in patients, reservoir hosts and vectors in Spain. PLoS One.

[52] Varanat M, Travis A, Lee W, Maggi RG, Bissett SA, Linder KE (2009). Recurrent osteomyelitis in a cat due to infection with *Bartonella vinsonii* subsp. *berkhoffii* genotype II. J Vet Intern Med.

[53] Ueno H, Hohdatsu T, Muramatsu Y, Koyama H, Morita C (1996). Does coinfection of *Bartonella henselae* and FIV induce clinical disorders in cats?. Microbiol Immunol.

[54] Glaus T, Hofmann-Lehmann R, Greene C, Glaus B, Wolfensberger C, Lutz H (1997). Seroprevalence of *Bartonella henselae* infection and correlation with disease status in cats in Switzerland. J Clin Microbiol.

[55] Lappin MR, Black JC (1999). *Bartonella* spp infection as a possible cause of uveitis in a cat. J Am Vet Med Assoc.

[56] Sykes JE, Westropp JL, Kasten RW, Chomel BB (2010). Association between *Bartonella* species infection and disease in pet cats as determined using serology and culture. J Feline Med Surg.

[57] Guptill L, Slater L, Wu CC, Lin TL, Glickman LT, Welch DF (1997). Experimental infection of young specific pathogen-free cats with Bartonella henselae. J Infect Dis.

[58] Kordick DL, Breitschwerdt EB (1997). Relapsing bacteremia after blood transmission of *Bartonella henselae* to cats. Am J Vet Res.

[59] O’Reilly KL, Bauer RW, Freeland RL, Foil LD, Hughes KJ, Rohde KR (1999). Acute clinical disease in cats following infection with a pathogenic strain of *Bartonella henselae* (LSU16). Infect Immun.

[60] Guptill L, Slater LN, Wu CC, Lin TL, Glickman LT, Welch DF (1998). Evidence of reproductive failure and lack of perinatal transmission of *Bartonella henselae* in experimentally infected cats. Vet Immunol Immunopathol.

[61] Perez C, Hummel JB, Keene BW, Maggi RG, Diniz PP, Breitschwerdt EB (2010). Successful treatment of *Bartonella henselae* endocarditis in a cat. J Feline Med Surg.

[62] Chomel BB, Wey AC, Kasten RW, Stacy BA, Labelle P (2003). Fatal case of endocarditis associated with *Bartonella henselae* type I infection in a domestic cat. J Clin Microbiol.

[63] Kordick DL, Hilyard EJ, Hadfield TL, Wilson KH, Steigerwalt AG, Brenner DJ (1997). *Bartonella clarridgeiae*, a newly recognized zoonotic pathogen causing inoculation papules, fever, and lymphadenopathy (cat scratch disease). J Clin Microbiol.

[64] La VD, Tran-Hung L, Aboudharam G, Raoult D, Drancourt M (2005). *Bartonella quintana* in domestic cat. Emerg Infect Dis.

[65] Breitschwerdt EB, Maggi RG, Sigmon B, Nicholson WL (2007). Isolation of *Bartonella quintana* from a woman and a cat following putative bite transmission. J Clin Microbiol.

[66] Chomel BB, Mac Donald KA, Kasten RW, Chang CC, Wey AC, Foley JE (2001). Aortic valve endocarditis in a dog due to *Bartonella clarridgeiae*. J Clin Microbiol.

[67] Breitschwerdt EB, Atkins CE, Brown TT, Kordick DL, Snyder PS (1999). *Bartonella vinsonii* subsp. *berkhoffii* and related members of the alpha subdivision of the Proteobacteria in dogs with cardiac arrhythmias, endocarditis, or myocarditis. J Clin Microbiol.

[68] Breitschwerdt EB, Kordick DL, Malarkey DE, Keene B, Hadfield TL, Wilson K (1995). Endocarditis in a dog due to infection with a novel *Bartonella* subspecies. J Clin Microbiol.

[69] Kordick DL, Swaminathan B, Greene CE, Wilson KH, Whitney AM, O’Connor S (1996). *Bartonella vinsonii* subsp. *berkhoffii* subsp. nov., isolated from dogs; *Bartonella vinsonii* subsp. *vinsonii* and emended description of *Bartonella vinsonii*. Int J Syst Bacteriol.

[70] Pappalardo BL, Brown T, Gookin JL, Morrill CL, Breitschwerdt EB (2000). Granulomatous disease associated with *Bartonella* infection in 2 dogs. J Vet Int Med.

[71] Kitchell BE, Fan TM, Kordick D, Breitschwerdt EB, Wollenberg G, Lichtensteiger CA (2000). Peliosis hepatis in a dog infected with *Bartonella henselae*. J Am Vet Med Assoc.

[72] Perez C, Maggi RG, Diniz PP, Breitschwerdt EB (2011). Molecular and serological diagnosis of *Bartonella* infection in 61 dogs from the United States. J Vet Int Med.

[73] Breitschwerdt EB, Linder KL, Day MJ, Maggi RG, Chomel BB, Kempf V (2013). Koch’s postulates and the pathogenesis of comparative infectious disease causation associated with *Bartonella* species. J Comp Pathol.

[74] Solano-Gallego L, Bradley J, Hegarty B, Sigmon B, Breitschwerdt E (2004). *Bartonella henselae* IgG antibodies are prevalent in dogs from southeastern USA. Vet Res.

[75] Duncan AW, Maggi RG, Breitschwerdt EB (2007). *Bartonella* DNA in dog saliva. Emerg Infect Dis.

[76] Gundi VA, Bourry O, Davous B, Raoult D, La Scola B (2004). *Bartonella clarridgeiae* and *B. henselae* in dogs, Gabon. Emerg Infect Dis.

[77] MacDonald KA, Chomel BB, Kittleson MD, Kasten RW, Thomas WP, Pesavento P (2004). A prospective study of canine infective endocarditis in northern California (1999-2001): emergence of *Bartonella* as a prevalent etiologic agent. J Vet Intern Med.

[78] Millar B, Moore J, Mallon P, Xu J, Crowe M, Mcclurg R (2001). Molecular diagnosis of infective endocarditis - a new Duke’s criterion. Scand J Infect Dis.

[79] Raoult D, Fournier PE, Drancourt M, Marrie TJ, Etienne J, Cosserat J (1996). Diagnosis of 22 new cases of *Bartonella* endocarditis. Ann Intern Med.

[80] Fournier PE, Lelievre H, Eykyn SJ, Mainardi JL, Marrie TJ, Bruneel F (2001). Epidemiologic and clinical characteristics of *Bartonella quintana* and *Bartonella henselae* endocarditis: a study of 48 patients. Med Chem Commun.

[81] Chang CC, Chomel BB, Kasten RW, Heller RM, Kocan KM, Ueno H (2000). *Bartonella* spp. isolated from wild and domestic ruminants in North America. Emerg Infect Dis.

[82] Chang C, Yamamoto K, Chomel BB, Kasten RW, Simpson DC, Smith CR (1999). Seroepidemiology of *Bartonella vinsonii* subsp. *berkhoffii* infection in California coyotes, 1994-1998. Emerg Infect Dis.

[83] Heller R, Kubina M, Mariet P, Riegel P, Delacour G, Dehio C (1999). *Bartonella alsatica* sp. nov., a new *Bartonella* species isolated from the blood of wild rabbits. Int J Syst Bacteriol.

[84] Chang CC, Hayashidani H, Pusterla N, Kasten RW, Madigan JE, Chomel BB (2002). Investigation of *Bartonella* infection in ixodid ticks from California. Comp Immunol Microbiol Infect Dis.

[85] Birtles RJ, Harrison TG, Molyneux DH (1994). *Grahamella* in small woodland mammals in the U.K.: isolation, prevalence and host specificity. Ann Trop Med Parasitol.

[86] Birtles RJ, Canales J, Ventosilla P, Alvarez E, Guerra H, Llanos-Cuentas A (1999). Survey of *Bartonella* species infecting intradomicillary animals in the Huayllacallan Valley, Ancash, Peru, a region endemic for human bartonellosis. Am J Trop Med Hyg.

[87] Ellis BA, Regnery RL, Beati L, Bacellar F, Rood M, Glass GG (1999). Rats of the genus *Rattus* are reservoir hosts for pathogenic *Bartonella* species: an Old World origin for a New World disease?. J Infect Dis.

[88] Fichet-Calvet E, Jomaa I, Ben IR, Ashford RW (2000). Patterns of infection of haemoparasites in the fat sand rat, *Psammomys obesus*, in Tunisia, and effect on the host. Ann Trop Med Parasitol.

[89] Heller R, Riegel P, Hansmann Y, Delacour G, Bermond D, Dehio C (1998). *Bartonella tribocorum* sp. nov., a new *Bartonella* species isolated from the blood of wild rats. Int J Syst Bacteriol.

[90] Hofmeister EK, Kolbert CP, Abdulkarim AS, Magera JM, Hopkins MK, Uhl JR (1998). Cosegregation of a novel *Bartonella* species with *Borrelia burgdorferi* and *Babesia microti* in *Peromyscus leucopus*. J Infect Dis.

[91] Kosoy MY, Regnery RL, Tzianabos T, Marston EL, Jones DC, Green D (1997). Distribution, diversity, and host specificity of *Bartonella* in rodents from the Southeastern United States. Am J Trop Med Hyg.

[92] Veikkolainen V, Vesterinen EJ, Lilley TM, Pulliainen AT. Bats as reservoir hosts of human bacterial pathogen, *Bartonella **mayotimonensis* Emerg Infect Dis. 2014;20:960–7. 10.3201/eid2006.130956 PMC4036794 24856523

[93] Hercik K, Hasova V, Janecek J, Branny P (2007). Molecular evidence of *Bartonella* DNA in ixodid ticks in Czechia. Folia Microbiol.

[94] Ihler GM (1996). *Bartonella bacilliformis*: dangerous pathogen slowly emerging from deep background. FEMS Microbiol Lett.

[95] Raoult D, Roux V (1999). The body louse as a vector of reemerging human diseases. Clin Infect Dis.

[96] Billeter SA, Levy MG, Chomel BB, Breitschwerdt EB (2008). Vector transmission of *Bartonella* species with emphasis on the potential for tick transmission. Med Vet Entomol.

[97] Brouqui P, Raoult D (2006). Arthropod-borne diseases in homeless. Ann N Y Acad Sci.

[98] Townsend CHT. Resumen de la labores en el Peru sobre el *Phlebotomus verrucarum* y su agencia en la transmission de la verruga. Annales Zoologici Aplicada. 1914;44.

[99] Brouqui P, Lascola B, Roux V, Raoult D (1999). Chronic *Bartonella quintana* bacteremia in homeless patients. N Engl J Med.

[100] Spach DH, Kanter AS, Dougherty MJ, Larson AM, Coyle MB, Brenner DJ (1995). *Bartonella* (*Rochalimaea*) *quintana* bacteremia in inner-city patients with chronic alcoholism. N Engl J Med.

[101] Maggi RG, Kempf VAJ, Chomel BB, Breitschwerdt EB. *Bartonella.* In: Versalovic J, Jorgensen JH, Funke G, Warnock DW, Landry ML, Carroll KC. Manual of Clinical Microbiology, 10th Edition. Washington DC: American Society of Microbiology; 2011. p. 786

[102] Chomel BB, Kasten RW, Williams C, Wey AC, Henn JB, Maggi R (2009). *Bartonella* endocarditis: a pathology shared by animal reservoirsand patients. Ann N Y Acad Sci.

[103] Lamas CDC, Ramos RG, Lopes GQ, Santos MS, Golebiovski WF, Weksler C (2013). *Bartonella* and *Coxiella* infective endocarditis in Brazil: Molecular evidence from excised valves from a cardiac surgery referral center in Rio de Janeiro, Brazil, 1998 to 2009. Int J Infect Dis.

[104] Tsukahara M, Tsuneoka H, Iino H, Murano I, Takahashi H, Uchida M (2000). *Bartonella henselae* infection as a cause of fever of unknown origin. J Clin Microbiol.

[105] Guiyedi V, Haddad H, Okome-Nkoumou M, Gire F, Ongali B, Lore P (2013). Cat-scratch disease in adult hospitalized for prolonged-Fever associated with multiple lymphadenopathies and weight loss. Open Microbiol J.

[106] Hadfield TL, Warren R, Kass M, Brun E, Levy C (1993). Endocarditis caused by Rochalimaea henselae. Hum Pathol.

[107] Spach DH, Callis KP, Paauw DS, Houze YB, Schoenknecht FD, Welch DF (1993). Endocarditis caused by *Rochalimaea quintana* in a patient infected with human immunodeficiency virus. J Clin Microbiol.

[108] Daly JS, Worthington MG, Brenner DJ, Moss CW, Hollis DG, Weyant RS (1993). *Rochalimaea elizabethae* sp. nov. isolated from a patient with endocarditis. J Clin Microbiol.

[109] Roux V, Eykyn SJ, Wyllie S, Raoult D (2000). *Bartonella vinsonii* subsp. *berkhoffii* as an agent of afebrile blood culture-negative endocarditis in a human. J Clin Microbiol.

[110] Fenollar F, Sire S, Raoult D (2005). *Bartonella vinsonii* subsp. *arupensis* as an agent of blood culture-negative endocarditis in a human. J Clin Microbiol.

[111] Raoult D, Roblot F, Rolain JM, Besnier JM, Loulergue J, Bastides F (2006). First isolation of *Bartonella alsatica* from a valve of a patient with endocarditis. J Clin Microbiol.

[112] Kosoy M, Murray M, Gilmore RD, Bai Y, Gage KL (2003). *Bartonella* strains from ground squirrels are identical to *Bartonella washoensis* isolated from a human patient. J Clin Microbiol.

[113] Jeanclaude D, Godmer P, Leveiller D, Pouedras P, Fournier PE, Raoult D (2009). *Bartonella alsatica* endocarditis in a French patient in close contact with rabbits. Clin Microbiol Infect.

[114] Lin EY, Tsigrelis C, Baddour LM, Lepidi H, Rolain JM, Patel R (2010). *Candidatus Bartonella mayotimonensis* and Endocarditis. Emerg Infect Dis.

[115] La Scola B, Raoult D (1999). Culture of *Bartonella quintana* and *Bartonella henselae* from human samples: a 5-year experience (1993 to 1998). J Clin Microbiol.

[116] Fournier PE, Mainardi JL, Raoult D (2002). Value of microimmunofluorescence for diagnosis and follow-up of *Bartonella* endocarditis. Clin Diagn Lab Immun.

[117] Maggi RG, Mozayeni BR, Pultorak EL, Hegarty BC, Bradley JM, Correa M (2012). *Bartonella* spp. bacteremia and rheumatic symptoms in patients from Lyme disease–endemic Region. Emerg Infect Dis.

[118] Breitschwerdt EB, Maggi RG, Lantos PM, Woods CW, Hegarty BC, Bradley JM (2010). *Bartonella vinsonii* subsp. *berkhoffii* and *Bartonella henselae* bacteremia in a father and daughter with neurological disease. Parasit Vectors.

[119] Maggi RG, Mascarelli PE, Pultorak EL, Hegarty BC, Bradley JM, Mozayeni BR (2011). *Bartonella* spp. bacteremia in high-risk immunocompetent patients. Diagn Microbiol Infect Dis.

[120] Halos L, Jamal T, Maillard R, Beugnet F, Le Menach A, Boulouis HJ (2005). Evidence of *Bartonella* sp. in questing adult and nymphal *Ixodes ricinus* ticks from France and co-infection with *Borrelia burgdorferi sensu lato* and *Babesia* sp. Vet Res.

[121] Eskow E, Rao RVS, Mordechai E (2001). Concurrent Infection of the Central Nervous System by *Borrelia burgdorferi* and *Bartonella henselae*. Arch Neurol.

[122] Podsiadly E, Chmielewski T, Tylewska-Wierzbanowska S (2003). *Bartonella henselae* and *Borrelia burgdorferi* infections of the central nervous system. Ann N Y Acad Sci.

[123] Holden K, Boothby JT, Kasten RW, Chomel BB (2006). Co-detection of *Bartonella henselae*, *Borrelia burgdorferi*, and *Anaplasma phagocytophilum* in *Ixodes pacificus* ticks from California, USA. Vector Borne Zoonotic Dis.

[124] Belongia EA (2002). Epidemiology and impact of coinfections acquired from *Ixodes* ticks. Vector Borne Zoonotic Dis.

[125] Lantos PM, Wormser GP (2014). Chronic coinfections in patients diagnosed with chronic Lyme disease: a systematic review. Am J Med.

[126] Lantos PM, Maggi RG, Ferguson B, Varkey J, Park LP, Breitschwerdt EB (2014). Detection of *Bartonella* species in the blood of veterinarians and veterinary technicians: a newly recognized occupational hazard?. Vector Borne Zoonotic Dis.

[127] Oliveira AM, Maggi RG, Woods CW, Breitschwerdt EB (2010). Suspected needle stick transmission of *Bartonella vinsonii* subspecies *berkhoffii* to a veterinarian. J Vet Intern Med.

[128] Breitschwerdt EB (2015). Did *Bartonella henselae* contribute to the deaths of two veterinarians?. Parasit Vectors.

[129] Jurke A, Bannert N, Brehm K, Fingerle V, Kempf VAJ, Kömpf D (2015). Serological survey of *Bartonella* spp., *Borrelia burgdorferi*, *Brucella* spp., *Coxiella burnetii*, *Francisella tularensis*, *Leptospira* spp., *Echinococcus*, Hanta-, TBE- and XMR-virus infection in employees of two forestry enterprises in North Rhine-Westphalia, Germany, 2011-2013. Int J Med Microbiol.

[130] Diniz PPV, Velho PENF, Pitassi LHU, Drummond MR, Lania BG, Barjas-Castro ML (2016). Risk Factors for *Bartonella* species Infection in Blood Donors from Southeast Brazil. PLoS Negl Trop Dis.

[131] Foil L, Andress E, Freeland RL, Roy AF, Rutledge R, Triche PC (1998). Experimental infection of domestic cats with *Bartonella henselae* by inoculation of *Ctenocephalides felis* (*Siphonaptera: Pulicidae*) feces. J Med Entomol.

[132] Chomel BB, Kasten RW (2010). Bartonellosis, an increasingly recognized zoonosis. J Appl Microbiol.

[133] Finkelstein JL, Brown TP, O’Reilly KL, Wedincamp J, Foil LD (2002). Studies on the growth of *Bartonella henselae* in the cat flea (Siphonaptera: Pulicidae). J Med Entomol.

[134] Bouhsira E, Franc M, Boulouis HJ, Jacquiet P, Raymond-Letron I, Liénard E (2013). Assessment of Persistence of *Bartonella henselae* in *Ctenocephalides felis*. Appl Environ Microbiol.

[135] Guptill L (2010). Bartonellosis. Vet Microbiol.

[136] Abbott RC, Chomel BB, Kasten RW, Floyd-Hawkins KA, Kikuchi Y, Koehler JE (1997). Experimental and natural infection with *Bartonella henselae* in domestic cats. Comp Immun Microbiol Infect Dis.

[137] Rolain JM, Franc M, Davoust B, Raoult D (2003). Molecular detection of *Bartonella quintana*, *B. koehlerae*, *B. henselae*, *B. clarridgeiae*, *Rickettsia felis*, and *Wolbachia pipientis* in cat fleas, France. Emerg Infect Dis.

[138] Sanogo YO, Zeaiter Z, Caruso G, Merola F, Shpynov S, Brouqui P (2003). *Bartonella henselae* in *Ixodes ricinus* ticks (*Acari: Ixodida*) removed from humans, Belluno province, Italy. Emerg Infect Dis.

[139] Durden LA, Ellis BA, Banks CW, Crowe JD, Oliver JH (2004). Ectoparasites of gray squirrels in two different habitats and screening of selected ectoparasites for bartonellae. J Parasitol.

[140] Bown KJ, Bennet M, Begon M (2004). Flea-borne *Bartonella grahamii* and *Bartonella taylorii* in bank voles. Emerg Infect Dis.

[141] Bermond D, Heller R, Barrat F, Delacour G, Dehio C, Alliot A (2000). Bartonella birtlesii sp. nov., isolated from small mammals (*Apodemus* spp.). Int J Syst Evol Microbiol.

[142] Reeves WK, Rogers TE, Dasch GA (2007). *Bartonella* and *Rickettsia* from fleas (*Siphonaptera: Ceratophyllidae*) of prairie dogs (*Cynomys* spp.) from the western United States. J Parasitol.

[143] Reeves WK, Rogers TE, Durden LA, Dasch GA (2007). Association of *Bartonella* with the fleas (*Siphonaptera*) of rodents and bats using molecular techniques. J Vector Ecol.

[144] Abbot P, Aviles AE, Eller L, Durden LA (2007). Mixed infections, cryptic diversity, and vector-borne pathogens: evidence from *Polygenis* fleas and *Bartonella* species. Appl Environ Microbiol.

[145] Jackson LA, Spach DH (1996). Emergence of *Bartonella quintana* infection among homeless persons. Emerg Infect Dis.

[146] Regnery R, Tappero J (1995). Unraveling mysteries associated with cat-scratch disease, bacillary angiomatosis, and related syndromes. Emerg Infect Dis.

[147] Sasaki T, Poudel SK, Isawa H, Hayashi T, Seki N, Tomita T (2006). First molecular evidence of *Bartonella quintana* in *Pediculus humanus capitis* (*Phthiraptera: Pediculidae*), collected from Nepalese children. J Med Entomol.

[148] Reeves WK, Szumlas DE, Moriarity JR, Loftis AD, Abbassy MM, Helmy IM (2006). Louse-borne bacterial pathogens in lice (*Phthiraptera*) of rodents and cattle from Egypt. J Parasitol.

[149] Battistini TS. Estudios sobre la verruga peruana. La Acción Médica. 1929

[150] Battistini TS (1931). La verrue péruvienne: sa transmission par le Phlébotome. Revue Sud-Americaine de Médicine et de Chirurgie.

[151] Hertig M (1942). Phlebotomus and Carrion’s disease. Am J Trop Med Hyg.

[152] Rynkiewicz EC, Hemmerich C, Rusch DB, Fuqua C, Clay K (2015). Concordance of bacterial communities of two tick species and blood of their shared rodent host. Mol Ecol.

[153] Benson MJ, Gawronski JD, Eveleigh DE, Benson DR (2004). Intracellular symbionts and other bacteria associated with deer ticks (*Ixodes scapularis*) from Nantucket and Wellfleet, Cape Cod, Massachusetts. Appl Environ Microbiol.

[154] Steiner FE, Pinger RR, Vann CN, Grindle N, Civitello D, Clay K, et al. Infection and co-infection rates of *Anaplasma phagocytophilum* variants, *Babesia* spp., *Borrelia burgdorferi*, and the rickettsial endosymbiont in *Ixodes scapularis* (Acari: Ixodidae) from sites in Indiana, Maine, Pennsylvania, and Wisconsin. J Med Entomol. 2008;45:289–97. 10.1603/0022-2585(2008)45[289:iacroa]2.0.co;2 18402145

[155] Sun J, Liu Q, Lu L, Ding G, Guo J, Fu G (2008). Coinfection with four genera of bacteria (*Borrelia*, *Bartonella*, *Anaplasma*, and *Ehrlichia*) in *Haemaphysalis longicornis* and *Ixodes sinensis* ticks from China. Vector Borne Zoonotic Dis.

[156] Vayssier-Taussat M, Moutailler S, Michelet L, Devillers E, Bonnet S, Cheval J (2013). Next generation sequencing uncovers unexpected bacterial pathogens in ticks in western Europe. PLoS One.

[157] Angelakis E, Billeter SA, Breitschwerdt EB, Chomel BB, Raoult D. Potential for tick-borne bartonelloses. Emerg Infect Dis. 2010;16:385–91. 10.3201/eid1603.091685 PMC3322042 20202411

[158] Dietrich F, Schmidgen T, Maggi RG, Richter D, Matuschka FR, Vonthein R (2010). Prevalence of *Bartonella henselae* and *Borrelia burgdorferi sensu lato* DNA in *Ixodes ricinus* ticks in Europe. Appl Environ Microbiol.

[159] Schouls LM, van de Pol I, Rijpkema SG, Schot CS (1999). Detection and identification of *Ehrlichia*, *Borrelia burgdorferi sensu lato*, and *Bartonella* species in Dutch *Ixodes ricinus* ticks. J Clin Microbiol.

[160] Chang CC, Chomel BB, Kasten RW, Romano V, Tietze N (2001). Molecular evidence of *Bartonella* spp. in questing adult *Ixodes pacificus* ticks in California. J Clin Microbiol.

[161] Morozova OV, Cabello FC, Dobrotvorsky AK (2004). Semi-nested PCR detection of *Bartonella henselae* in *Ixodes persulcatus* ticks from Western Siberia, Russia. Vector Borne Zoonotic Dis.

[162] Kim CM, Kim JY, Yi YH, Lee MJ, Cho MR, Shah DH (2005). Detection of *Bartonella* species from ticks, mites and small mammals in Korea. J Vet Sci.

[163] Podsiadly E, Chmielewski T, Sochon E, Tylewska-Wierzbanowska S (2007). *Bartonella henselae* in *Ixodes ricinus* ticks removed from dogs. Vector Borne Zoonotic Dis.

[164] Adelson ME, Rao RVS, Tilton RC, Cabets K, Eskow E, Fein L (2004). Prevalence of *Borrelia burgdorferi*, *Bartonella* spp., *Babesia microti*, and *Anaplasma phagocytophila* in *Ixodes scapularis* ticks collected in Northern New Jersey. J Clin Microbiol.

[165] Janecek E, Mietze A, Goethe R, Schnieder T, Strube C (2012). *Bartonella* spp. infection rate and *B. grahamii* in ticks. Emerg Infect Dis.

[166] Reye AL, Hubschen JM, Sausy A, Muller CP (2010). Prevalence and seasonality of tick-borne pathogens in questing *Ixodes ricinus* ticks from Luxembourg. Appl Environ Microbiol.

[167] Sytykiewicz H, Karbowiak G, Werszko J, Czerniewicz P, Sprawka I, Mitrus J (2012). Molecular screening for *Bartonella henselae* and *Borrelia burgdorferi sensu lato* co-existence within *Ixodes ricinus* populations in central and eastern parts of Poland. Ann Agric Environ Med.

[168] Moutailler S, Valiente Moro C, Vaumourin E, Michelet L, Tran FH, Devillers E, et al. Co-infection of ticks: The rule rather than the exception. PLoS Negl Trop Dis. 2016;10:e0004539. 10.1371/journal.pntd.0004539 PMC4795628 26986203

[169] Sormunen JJ, Penttinen R, Klemola T, Hanninen J, Vuorinen I, Laaksonen M (2016). Tick-borne bacterial pathogens in southwestern Finland. Parasit Vectors.

[170] Müller A, Reiter M, Schötta AM, Stockinger H, Stanek G. Detection of *Bartonella* spp. in *Ixodes ricinus* ticks and *Bartonella* seroprevalence in human populations. Ticks Tick Borne Dis. 2016. doi:10.1016/j.ttbdis.2016.03.009. 10.1016/j.ttbdis.2016.03.009 26997137

[171] Cotté V, Bonnet S, Le Rhun D, Le Naour E, Chauvin A, Boulouis HJ (2008). Transmission of *Bartonella henselae* by *Ixodes ricinus*. Emerg Infect Dis.

[172] Reis C, Cote M, Le Rhun D, Lecuelle B, Levin ML, Vayssier-Taussat M (2011). Vector competence of the tick *Ixodes ricinus* for transmission of *Bartonella birtlesii*. PLoS Negl Trop Dis.

[173] Podsiadly E, Karbowiak G, Tylewska‐Wierzbanowska S (2009). Presence of *Bartonella* spp. in *Ixodidae* ticks. Clin Microbiol Infect.

[174] Tuttle AD, Birkenheuer AJ, Juopperi T, Levy MG, Breitschwerdt EB (2003). Concurrent bartonellosis and babesiosis in a dog with persistent thrombocytopenia. J Am Vet Med Assoc.

[175] Pappalardo BL, Correa MT, York CC, Peat CY, Breitschwerdt EB (1997). Epidemiologic evaluation of the risk factors associated with exposure and seroreactivity to *Bartonella vinsonii* in dogs. Am J Vet Res.

[176] Breitschwerdt EB, Hegarty BC, Hancock SI (1998). Sequential evaluation of dogs naturally infected with *Ehrlichia canis*, *Ehrlichia chaffeensis*, *Ehrlichia equi*, *Ehrlichia ewingii*, or *Bartonella vinsonii*. J Clin Microbiol.

[177] Honadel TE, Chomel BB, Yamamoto K, Chang C, Farver TB (2001). Seroepidemiology of *Bartonella vinsonii* subsp *berkhoffii* exposure among healthy dogs. J Am Vet Med Assoc.

[178] Kordick SK, Breitschwerdt EB, Hegarty BC, Southwick KL, Colitz CM, Hancock SI (1999). Coinfection with multiple tick-borne pathogens in a Walker Hound kennel in North Carolina. J Clin Microbiol.

[179] Morozova OV, Chernousova N, Morozov IV (2005). Detection of the *Bartonella* DNA by the method of nested PCR in patients after tick bites in Novosibirsk region. Mol Gen Mikrobiol Virusol.

[180] Vayssier-Taussat M, Moutailler S, Féménia F, Raymond P, Croce O, La Scola B (2016). Identification of Novel Zoonotic Activity of *Bartonella* spp., France. Emerg Infect Dis.

[181] Lucey D, Dolan MJ, Moss CW, Garcia M, Hollis DG, Wegner S (1992). Relapsing illness due to *Rochalimaea henselae* in immunocompetent hosts: implication for therapy and new epidemiological associations. Clin Infect Dis.

[182] van Buskirk J, Ostfeld RS (1995). Controlling Lyme-disease by modifying the density and species composition of tick hosts. Ecol Appl.

[183] Keesing F, Holt RD, Ostfeld RS (2006). Effects of species diversity on disease risk. Ecol Lett.

[184] Telford SR, Wormser GP (2010). *Bartonella* spp. transmission by ticks not established. Emerg Infect Dis.

[185] Chung CY, Kasten RW, Paff SM, van Horn BA, Vayssier-Taussat M, Boulouis HJ (2004). *Bartonella* spp. DNA associated with biting flies from California. Emerg Infect Dis.

[186] Reeves WK, Dowling AP, Dasch GA. Rickettsial agents from parasitic *dermanyssoidea* (Acari: Mesostigmata). Exp Appl Acarol. 2006;38:181–8. 10.1007/s10493-006-0007-1 16596351

[187] Dehio C (2004). Molecular and cellular basis of bartonella pathogenesis. Annu Rev Microbiol.

[188] Reeves WK, Nelder MP, Cobb KD, Dasch GA. *Bartonella* spp. in deer keds, *Lipoptena mazamae* (Diptera: Hippoboscidae), from Georgia and South Carolina, USA. J Wildl Dis. 2006;42:391–6. 10.7589/0090-3558-42.2.391 16870863

[189] Matsumoto K, Berrada ZL, Klinger E, Goethert HK, Telford SR (2008). Molecular detection of *Bartonella schoenbuchensis* from ectoparasites of deer in Massachusetts. Vector Borne Zoonotic Dis.

[190] Dehio C, Sauder U, Hiestand R (2004). Isolation of *Bartonella schoenbuchensis* from *Lipoptena cervi*, a blood-sucking arthropod causing deer ked dermatitis. J Clin Microbiol.

[191] Halos L, Jamal T, Maillard R, Girard B, Guillot J, Chomel B (2004). Role of *Hippoboscidae* flies as potential vectors of *Bartonella* spp. infecting wild and domestic ruminants. Appl Environ Microbiol.

