# Erratum to: *Bartonella* spp. - a chance to establish One Health concepts in veterinary and human medicine

**DOI:** 10.1186/s13071-016-1604-4

**Published:** 2016-06-10

**Authors:** Yvonne Regier, Fiona O’Rourke, Volkhard A. J. Kempf

**Affiliations:** Institute for Medical Microbiology and Infection Control, University Hospital, Goethe-University, Frankfurt am Main, Germany

## Erratum

Unfortunately, the original version of this article [1] contained an error. The name of the author Fiona O’Rourke was incorrectly spelt as Fiona O´Rourke. The correct spelling is included here, and has been updated in the original article.
